# A nanonewton-scale biomimetic mechanosensor

**DOI:** 10.1038/s41378-023-00560-w

**Published:** 2023-07-11

**Authors:** Chi Zhang, Mengxi Wu, Ming Li, Lixuan Che, Zhiguang Tan, Di Guo, Zhan Kang, Shuye Cao, Siqi Zhang, Yu Sui, Jining Sun, Liding Wang, Junshan Liu

**Affiliations:** 1 State Key Laboratory of High-performance Precision Manufacturing, Dalian University of Technology, 116024 Dalian, Liaoning China; 2 Key Laboratory for Micro/Nano Technology and System of Liaoning Province, Dalian University of Technology, 116024 Dalian, Liaoning China; 3 State Key Laboratory of Structural Analysis for Industrial Equipment, Dalian University of Technology, 116024 Dalian, China

**Keywords:** Electrical and electronic engineering, Electronic properties and materials

## Abstract

Biomimetic mechanosensors have profound implications for various areas, including health care, prosthetics, human‒machine interfaces, and robotics. As one of the most important parameters, the sensitivity of mechanosensors is intrinsically determined by the detection resolution to mechanical force. In this manuscript, we expand the force detection resolution of current biomimetic mechanosensors from the micronewton to nanonewton scale. We develop a nanocrack-based electronic whisker-type mechanosensor that has a detection resolution of 72.2 nN. We achieve the perception of subtle mechanical stimuli, such as tiny objects and airflow, and the recognition of surface morphology down to a 30 nm height, which is the finest resolution ever reported in biomimetic mechanosensors. More importantly, we explore the use of this mechanosensor in wearable devices for sensing gravity field orientation with respect to the body, which has not been previously achieved by these types of sensors. We develop a wearable smart system for sensing the body’s posture and movements, which can be used for remote monitoring of falls in elderly people. In summary, the proposed device offers great advantages for not only improving sensing ability but also expanding functions and thus can be used in many fields not currently served by mechanosensors.

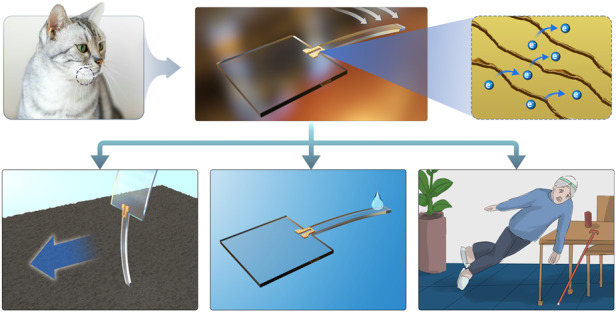

## Introduction

Animals can perceive various types of mechanical stimuli that come from the external environment by using their natural sensors. For example, skin, whiskers, cochlea, and vestibules can detect touch, track fluid motions, sense acoustic waves, and navigate a gravity field, respectively^1^. Although these sensors seem to be quite different, they can be intrinsically concluded to be one type of sensor, a mechanosensor, which perceives a mechanical force induced by the external environment and applied to the sensing element. Decoding these sensing mechanisms and learning from natural mechanosensors have greatly inspired people to develop biomimetic devices for artificial sensing technology^2^. In recent years, tremendous efforts have been devoted to this field, with a vast number of publications continuously reporting exciting results. To date, a great variety of devices have been developed for mapping tactility^3–6^, detecting objects in the surrounding environment^7,8^, recognizing surface morphology^9–11^, feeling airflows^12–14^, sensing vibrations or sound waves^15–17^, and so on. These mechanosensors offer the potential of profound implications for various areas, including but not limited to prosthetics, health care, human‒machine interactions, and robotics^18–20^.

For all these biomimetic mechanosensors, sensitivity is one of the most important parameters in evaluating device performance. Despite the different characterization forms, the sensitivity of a biomimetic mechanosensor is essentially determined by the force detection resolution since the underlying physical quantity measured by the sensing element is the force. The force detection resolution is the minimum value of the force that a sensor can distinguish. Currently, many biomimetic mechanosensors have demonstrated the ability to detect subtle forces, and the detection resolution ranges from 0.1 to 18 mN^21–24^. In other studies, the detection resolution has ranged from 0.9 to 5 Pa in terms of pressure^13,15,24–27^. The equilibrium force detection resolution is on the submillinewton to millinewton scale after converting pressure to force. Most recently, by utilizing a triboelectric nanogenerator, An et al. developed an electronic whisker-type mechanosensor that could distinguish an exciting force to the level of 1.129 μN^7^. This work is a tremendous advancement that encourages the exploration of mechanosensors with superfine resolution. Therefore, after successfully achieving micronewton scale detection resolution, the next milestone for the mechanosensor is to further improve the force detection resolution to the nanonewton scale.

However, it is very challenging for biomimetic mechanosensors to detect nanonewton scale force. In fact, even though the constraints required by practical applications such as wearable devices or flexible humanoid robots are not considered, the detection of nanonewton scale force is not easy to achieve. It requires bulky instruments such as atomic force microscopy (AFM), which requires complicated control and detection systems for support. Some studies have reported sensitivity to the nanonewton scale by utilizing silicone-based devices^28–30^. However, these devices require intensive MEMS fabrication ability and are expensive. The lack of a simple, cost-effective, and wearable device with nanonewton-scale detection resolution hinders further advancements in the applications of biomimetic mechanosensors in many fields.

**Fig. 1:**
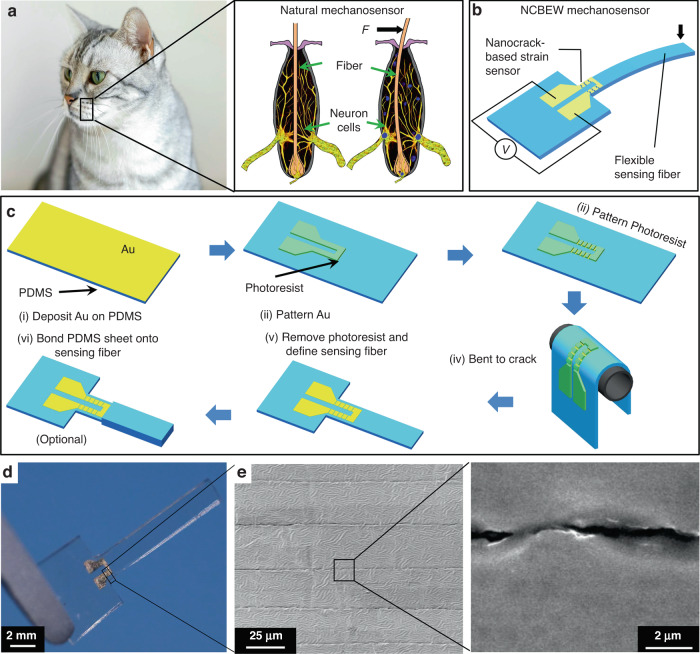
The nanocrack-based electronic whisker mechanosensor. **a** Schematic illustration of the NCBEW mechanosensor and its biomimetic comparison with the cat whisker sensory system. **b** Schematic of the NCBEW mechanosensor. **c** The manufacturing process of the NCBEW mechanosensor. **d** Photograph of an NCBEW mechanosensor. **e** SEM images of the nanocracks in the Au layer with the ordered arrangement

To resolve this obstacle, in this manuscript, we introduce efforts to develop an ultrahighly sensitive biomimetic mechanosensor. First, we imitate the principle and structure of mammalian whiskers to construct a whisker-type mechanosensor consisting of a flexible sensing fiber to undertake and transfer external mechanostimuli to the internal strain of the structure. Additionally, we implement a nanocrack-based strain sensor to transfer internal strain to output electrical signals. A nanocrack-based strain sensor that imitates the mechanism that spiders use to sense minute variations was first reported by Kang et al.^31^. However, the precise manufacturing of nanocracks on a flexible and small sensing fiber is difficult, thus preventing researchers from using nanocrack-based strain sensors in whisker-type devices. Recently, we developed a photolithography-assisted method that can precisely fabricate patterned nanocracks at designated positions^32^. Therefore, we are able to construct an ultrahighly sensitive mechanosensor utilizing a nanocrack-based electronic whisker (NCBEW), as shown in Fig. 1.

We characterize the NCBEW mechanosensor, and it can achieve force detection resolution down to 72.2 nN, which is the first biomimetic mechanosensor with nanonewton-scale detection resolution. The improvement in sensitivity yields the ability to perceive subtle mechanical stimuli, which is essential in many circumstances to track minor changes in the external environment. We demonstrate the perception of tiny objects such as a single section of hair, the feeling of airflow induced by breathing or a door’s opening and closing. We also use the NCBEW mechanosensor to recognize surface morphology and achieve the perception of a 30 nm-height step, which is also the finest resolution ever reported regarding biomimetic mechanosensors. Furthermore, since the NCBEW mechanosensor is so sensitive that it can recognize the torsion induced by its own gravity, we demonstrate the sensing of the tilted angle of a body with respect to the Earth’s vertical. A fully integrated and wearable hairband has been developed based on the NCBEW device for real-time monitoring of human body posture and movements such as sleeping position and falling, which mimics the functions of the human vestibule.

In summary, the proposed NCBEW mechanosensor expands the force detection resolution of current biomimetic mechanosensors from the micronewton to nanonewton scale. It is reasonably believed that it not only endows devices with more sensitive sensing ability to the surrounding environment but also provides an opportunity for personal health care and potential applications such as artificial vestibules for gravity perception.

## Results

### Principle of the NCBEW mechanosensor

The NCBEW mechanosensor imitates the whisker sensory system of animals. The whiskers of a cat and the underlying sensing mechanism are illustrated in Fig. 1a. Specifically, a whisker fiber grows in the hair follicle with its root being surrounded by neuro cells. When a mechanical stimulus such as an external force is applied to the fiber, the deformation of the fiber pushes or pulls the neuron cells due to its bending. Then, neuronal cells transfer the mechanical stimulus to electric signals and finally send the electric signals to the brain^33,34^. As shown in Fig. 1b, we propose to use a polydimethylsiloxane (PDMS) cantilever as the flexible sensing fiber to imitate the whisker fiber that undertakes and transfers the external load to internal strain and a U-shaped nanocrack-based strain sensor located at the root area of the cantilever to function as neuron cells that sense the deformation of the sensing fiber and generate electrical signals.

The manufacturing process of the NCBEW mechanosensor is shown in Fig. 1c. First, a layer of Au was sputtered onto a PDMS sheet. Second, Au was patterned by using standard photolithography. Third, the photoresist on the Au was patterned again to define the location of nanocracks. Fourth, the sample was bent to generate cracks. Fifth, the photoresist of the sample was removed, and the PDMS sheet was defined as a sensing fiber by using a knife die. Optionally, for tilt angle sensing, two pieces of PDMS were bonded onto the two sides of the sensing fiber (The detailed information is described in Note S1 of the Supplementary information.). Figure 1d shows a photograph of an NCBEW mechanosensor. Figure 1e shows the nanocracks precisely manufactured in the Au layer, and the overlapped crack edges can be observed.

**Fig. 2:**
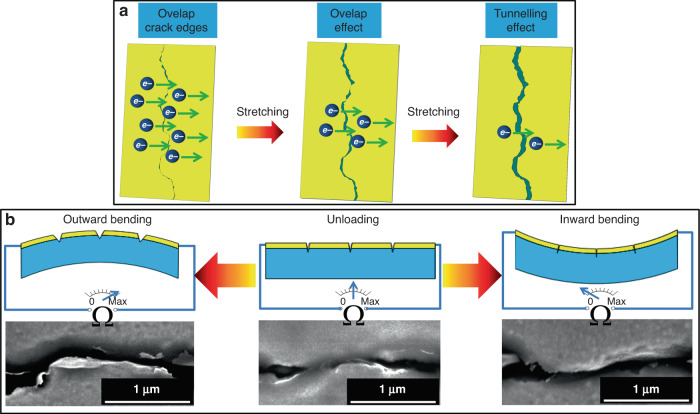
Schematic diagram of the working principle of the nanocrack-based strain sensing unit. **a** The resistance change of a nanocrack when the crack opens. **b** Nanocrack-based strain sensing unit percepts the bending of the flexible sensing fiber

An illustration of a nanocrack-based strain sensing unit is shown in Fig. 2a. The working principle of the Au/PDMS nanocrack-based strain sensing unit includes the overlap effect and the tunneling effect^35,36^. Therefore, when a mechanical stimulus is applied to the sensing fiber that forces the fiber to bend outward, the electrode is stretched, which makes nanocracks open. As a result, the resistance of the Au layer increases dramatically, therefore indicating the value of the applied mechanical stimulus (Fig. 2b). In contrast, when the sensing fiber is forced to bend inward, the crack edges overlap, and the resistance of the Au layer decreases. In addition, combining the previous reports involving nanocrack-based sensors^35,36^, an electromechanical model is established in Note S2 (Supplementary information) to show the relationship between the output of the proposed NCBEW mechanosensor and the applied force.

### Characterization of the NCBEW mechanosensor

**Fig. 3:**
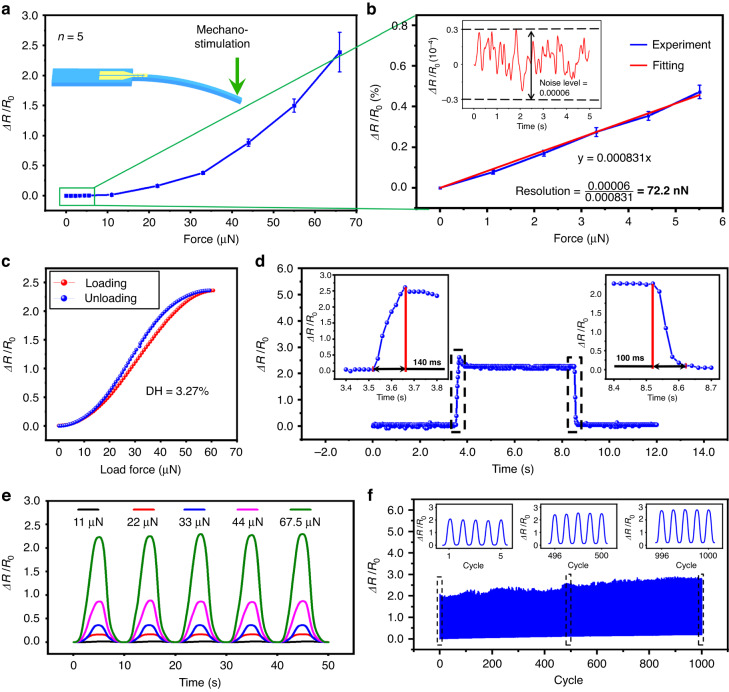
Characterization of the NCBEW mechanosensor. **a** The NCBEW mechanosensor responds to the applied force with a wide range from 0 to 67.5 μN. **b** The linear relationship between the applied force and relative resistance changes within the range from 0 to 5.5 μN. Considering the noise level, the minimum resolution for force detection is as low as 72.2 nN. **c** The hysteresis behavior of the NCBEW device with a degree of hysteresis (DH) of 3.27%. **d** The response time and recovery time are 140 ms and 100 ms. **e** Repeatability is demonstrated via the response of the NCBEW device to periodic loading forces of 11 μN, 22 μN, 33 μN, 44 μN, and 67.5 μN. **f** Characterization of the durability of the NCBEW device with loading cycles up to 1000

The sensing performance of the NCBEW mechanosensor is investigated and shown in Fig. 3. The NCBEW mechanosensor exhibits ultrahigh sensitivity, including gauge factor (GF) and detection resolution. Figure 3a shows the response of the NCBEW mechanosensor to force in the range of 0–67.5 μN. Specifically, the NCBEW mechanosensor responds to the loading force of 67.5 μN with an Δ*R*/*R*_0_ of 2.39 ± 0.33 (the responses of five samples are shown in Fig. S1); therefore, the GF of the NCBEW mechanosensor is 682.85 ± 94.28 (the corresponding strain is 0.35%, and the detailed calculation is described as the model in the section of Note S3 in the Supplementary information). According to the proposed electromechanical model (Note S2, Supplementary information), we also conduct theoretical fitting of the response of the proposed NCBEW mechanosensor. As shown in Fig. S2, the proposed electromechanical model can fit the response of the proposed NCBEW mechanosensor. Additionally, the detection resolution of the NCBEW mechanosensor is on the nanonewton scale. As shown in Fig. 3b, within the range of 0–5.5 μN, a linear relationship is fitted with the Equation y = 0.000831x, and the coefficient of determination R^2^ is equal to 0.995. Referring to the method reported in the work done by Wang’s group^7^, we calculate the detection resolution by measuring the noise level for the output signal and dividing its value by the slope of the fitting equation. The resistance of the device when no force is loaded, as well as the fluctuation of the resistance, are measured. Then, the relative resistance change is calculated by dividing the fluctuation of the resistance by the average of the resistance and is defined as the noise level (the results are shown by the inserted figure in Fig. 3b). Considering the noise level to be 0.00006, the force detection resolution of the NCBEW device is as low as 72.2 nN. It is worth noting that the strain of the nanocrack sensing unit deciding the output of the device is reciprocally proportional to the flexural rigidity of the sensing fiber (Eq. (S1)). That is, the force detection resolution of the device could be improved using softer materials, thinner sensing fibers, etc. These results demonstrate that we can expand the force detection resolution of biomimetic mechanosensors from the micronewton level to the nanonewton scale.

We also perform thorough quantitative characterization by applying precisely controlled mechanical stimuli to the NCBEW device. Vertical deflections with specific parameters are applied on the free end of the sensing fiber using an electronic universal testing machine. The equivalent loading force can be calculated by the deflection of the flexible sensing fiber. The detailed information is described in Note S3 (Supplementary information). First, the hysteresis behavior of the device was studied by applying a gradually changing force. As shown in Fig. 3c, the response curves regarding the loading and unloading process nearly overlap. The degree of hysteresis (DH) is 3.27% according to a previously reported calculation method^37^. A square wave loading with a force amplitude of 67.5 μN was applied to the NCBEW device to check the response time and recovery time. As shown in Fig. 3d, the response time and recovery time are 140 ms and 100 ms, respectively.

Figure 3e demonstrates the repeatability of the NCBEW mechanosensor. When varied mechanical stimuli are applied periodically, the device generates signals correspondingly, and the signal amplitudes of all cycles remain constant. Durability, which is considered another important performance parameter for sensors, is tested. By applying a force of 67.5 μN at a loading frequency of 0.2 Hz to the NCBEW mechanosensor, the Δ*R*/*R*_0_ from the 1st cycle to the 1000th cycle is presented in Fig. 3f. The average maximum values of Δ*R*/*R*_0_ calculated based on the amplitudes of five cycles near the 500th and 1000th cycles are 2.35 and 2.56, respectively. Compared with the maximum value of Δ*R*/*R*_0_ (2.11) in the first cycle, the relative differences are 11.77% and 21.52%. The change in the response results from strain accumulation^38^.

The effects of temperature, humidity and packaging on the response of the NCBEW mechanosensor were also evaluated. As shown in Fig. S3a, when the temperature increases from 25 °C to 55 °C, the Δ*R*/*R*_0_ of the device increases from 0 to 1.08 ± 0.19 (data were obtained from 5 samples). These results show an obvious temperature coefficient due to the large coefficient of thermal expansion (CTE) of the PDMS substrate and the temperature resistance effect of Au^39^. To minimize the temperature coefficient, materials with a lower CTE, such as polyimide, can be used to replace PDMS, and algorithms that could eliminate the temperature resistance effect of Au should be further studied. Additionally, a differential design should be constructed in future studies. As shown in Fig. S3b, in addition to the U-shape nanocrack-based sensing unit at the fixed end, the differential design includes another U-shaped nanocrack-based sensing unit at the free end of the fiber where the strain is minimal. The second U-shaped nanocrack-based sensing unit serves as a temperature unit that can eliminate the effect of temperature on the response of the NCBEW mechanosensor. To evaluate the effect of humidity on the response of the sensor, the sensor was placed in an airtight environment where the humidity was changed by using a humidifier. The response of the process is recorded in Fig. S3c. When the relative humidity increases from 20% to 90%, there is little change caused by the change in humidity, which means that the sensor has good resistance to humidity change. We also investigate the effect of the packaging on the response of the NCBEW mechanosensor. The Δ*R*/*R*_0_ of the device after packaging is lower than that before packaging, and the GF of the packaged nanocrack-based strain sensing unit is 458.48 (Fig. S3d). Previous studies have shown a decrease in sensitivity to packaged nanocrack-based sensors^40–43^. On the other hand, the packaging material increases the thickness of the cantilever and encapsulates the nanocrack sensing unit inside the structure; therefore, the sensing unit is closer to the neutral layer of the cantilever structure so that less strain is applied when the same force is applied (Note S3, Fig. S3e, f).

### Tactile recognition and airflow monitoring

**Fig. 4:**
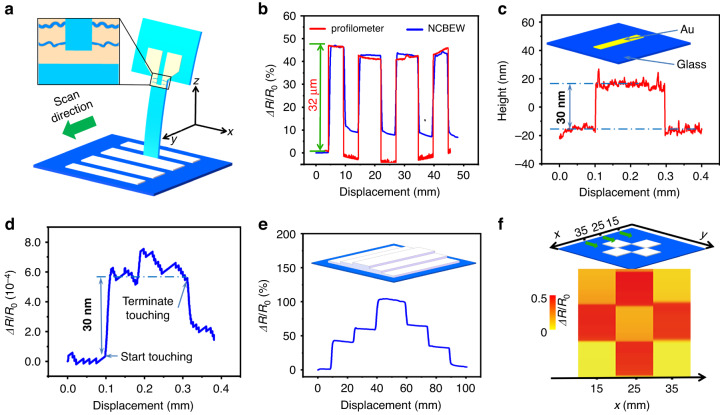
Applications of the NCBEW mechanosensor for tactile recognition. **a** Schematic of the NCBEW mechanosensor for tactile recognition. **b** The scanning results of the NCBEW mechanosensor to the samples with a stage height of 32 μm. The blue line and red line indicate the data obtained by the NCBEW device and surface profilometer, respectively. **c** The profiling results of a 30-nm-thick Au stripe on a glass obtained by surface profilometry and (**d**) the corresponding results obtained from the NCBEW mechanosensor. **e** Surface profiling results for a sample with three different heights. **f** The surface morphology mapping result for complex structures

The improvement of a mechanosensor’s sensitivity yields better performance and sensing ability in practical applications. To demonstrate the advantages of the NCBEW mechanosensor, we utilize our devices for tactile recognition and airflow monitoring. The results are shown in Fig. 4.

In nature, animals use skin or whiskers to perceive tactile sense, such as the roughness of the surface. By mounting the device on a movable robotic arm to touch and scan the surface, our NCBEW mechanosensor can be applied for tactile sensing (as shown in Figs. 4a, S4 of the Supplementary information). When the sensing fiber touches the patterned protruding features, it bends outward and leads to an increase in the resistance, while when the device crosses the bumps, the sensing fiber recovers, which results in a decrease in the resistance. Figure 4b shows the surface profiling results for continuously scanning patterned fringes. The testing sample contains four SU-8 photoresist fringes on the surface. The pattern has a spacing of 5 mm and a height of approximately 32 μm. The scanning results obtained from the NCBEW mechanosensor regarding the relative change in resistance (blue line) and the data obtained by the surface profilometer (red line) are compared. The two lines agree well with each other. Then, to perceive the nanometer-scale surface morphology, we develop an NCBEW device with an inhomogeneous cross-section that has a superior ability to recognize sensing fiber bending due to the concentration effect of strain (Fig. S5 and Note S4 in Supplementary information). Figure 4c shows an Au stripe with a height of 30 nm deposited on glass, and its surface is characterized via a commercial surface profilometer. Figure 4d shows the signal obtained from the NCBEW mechanosensor. A step change is recorded when the sensing fiber starts or terminates touching the Au stripe. The results demonstrate that the NCBEW mechanosensor is ultrasensitive for tactile perception and capable of perceiving nanometer-scale surface morphology, which is the finest resolution of all biomimetic mechanosensors to our knowledge^9,10,21,22,44,45^. In addition, we also test the NCBEW mechanosensor for tactile recognition of complex surface morphology. Figure 4e shows that three SU-8 stages with different heights are recognized. Figure 4f shows the mapping of the spatial distribution of four photoresist blocks. The surface morphology scanning results of the NCBEW mechanosensor match well with those obtained from a commercial surface profilometer, as shown in Fig. S6.

**Fig. 5:**
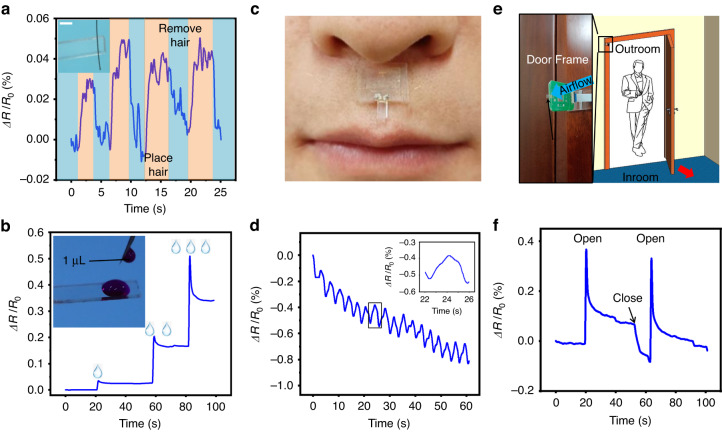
Applications of the NCBEW mechanosensor for tiny object and airflow monitoring. **a** The response of the NCBEW mechanosensor when weighing a section of hair 4 times. (The weight of the hair is ~ 0.1 μg. Scale bar: 2 mm.) **b** The response of the NCBEW device when water drops fall onto the sensing fiber. **c** Monitoring the airflow induced by human breath. **d** The response of the NCBEW mechanosensor records breathing in 1 min. **e** Monitoring the airflow induced by the opening or closing of the door. **f** The signals obtained by the NCBEW mechanosensor when opening and closing the door of a closed room

The NCBEW mechanosensor is capable of imitating mammal whiskers to sense minute changes in the environment. Figure 5a shows the ability to detect a superlight object. A section of human hair (the length of hair is ~1 cm and the weight is ~0.1 μg) is placed on the device’s end and then removed 4 times. The device’s output signal can distinguish the process clearly. Figure 5b shows the response of the NCBEW mechanosensor when water drops with a volume as small as 1 μL fall onto the device. The signal exhibits an overshoot due to the impulse of the water drop’s momentum and then stays at constant levels until another water drop falls.

Monitoring the airflow can obtain the speed and direction of the wind. Furthermore, the minute airflow disturbance is a sign of changes in the external environment, such as an object moving or approaching. The ability of the NCBEW mechanosensor to monitor the airflow is also characterized. We attach our device at the philtrum position to monitor the airflow caused by human breathing, as shown in Fig. 5c. Human breathing can reflect the state of personal health; for example, sleep apnea syndrome may lead to hypertension, coronary heart disease, and even sudden death^46^. To monitor human breathing, some flexible strain sensors were employed by attaching the flexible sensors onto the skin of the chest^47,48^. We provide a more convenient approach to monitor human breathing flow. As shown in Fig. 5d (and in Movie S1, Supplementary information), 16 peaks of the relative change in resistance reflect human breathing in one minute. We also demonstrate the use of our NCBEW mechanosensor to sense the opening and closing of the door by monitoring the corresponding airflow, as shown in Fig. 5e. When the door is opening or closing, the movement of the door panel induces positive or negative pressure, thus generating airflow disturbance. Figure 5f shows the response of the NCBEW mechanosensor to monitor the opening and closing of the door. These results indicate the ability of our device to track environmental changes via the perception of airflow.

### Perception of the body’s orientation with respect to the gravity field

**Fig. 6:**
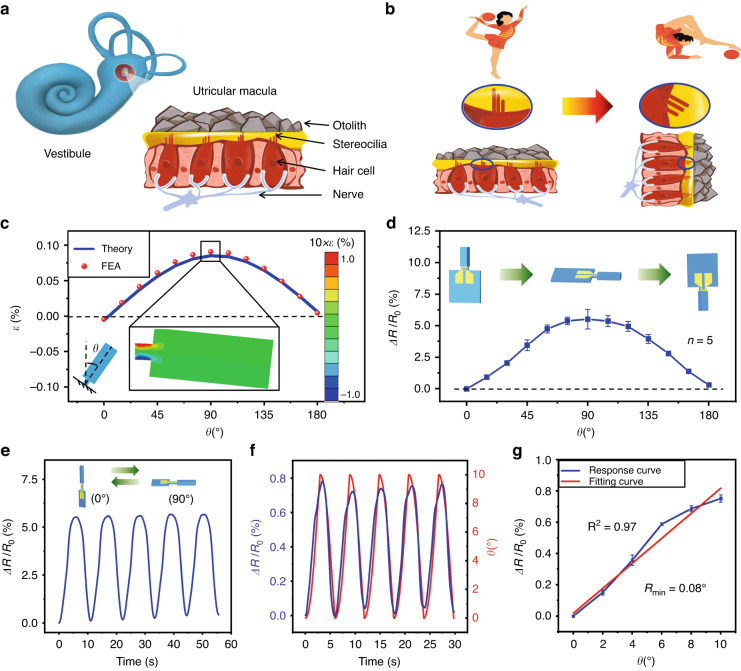
The NCBEW mechanosensor sensing tilt angle. **a** The structure of the utricular macula in the human vestibule. **b** Schematic of the vestibule’s function in terms of gravity perception. The posture of the body induces otoliths to be at different positions that drag stereocilia and stimulate hair cells to respond. **c** Theoretical and finite element analysis (FEA) of the strain as a function of the inclination angle of the NCBEW mechanosensor. **d** The response of the NCBEW mechanosensor to inclination angles ranging from 0° to 180°. **e** Repeatability tests by swinging the NCBEW device back and forth from 0° to 90° periodically. **f** The response of the NCBEW mechanosensor to a small tilted angle ranging from 0° to 10°. **g** Linear fitting of the relationship between the tilted angle and relative resistance change

In nature, humans and some animals use the vestibule system to sense Earth’s gravity, which acts as a pervasive environmental stimulus on the body. The vestibule system perceives gravity via the otolith and stereocilia (Fig. 6a, b). The sense of gravity is referred to as graviception, which is required for postural stability and spatial orientation recognition^49^. Inspired by the vestibule system, the NCBEW mechanosensor can perceive the torsion force induced by the sensing fiber’s own gravity due to the superfine force detection resolution. When the device rotates, the tilted angle between the direction of the sensor’s body and the direction of the gravity field changes; therefore, the torsion force applied to the sensing fiber changes. By recognizing the difference in torsion force, our device can sense the orientation of the device itself. A detailed analysis is described in Note S5 and Fig. S7 in the Supplementary information. We define the inclination angle *θ* as the angle between the vertically upward direction and the direction of the sensing fiber. The relationship between the value of strain at the root part of the sensing fiber and inclination angle *θ* is investigated via a theoretical model and finite element analysis (FEA). The results are plotted in Fig. 6c. Specifically, the strain increases when *θ* changes from 0° to 90°, while it decreases when *θ* changes from 90° to 180°. The maximum strain is ~0.08% when the sensing fiber is in a horizontal position. Figure 6d shows the results in terms of the NCBEW mechanosensor’s response when *θ* changes from 0° to 180°. The output signal presented as Δ*R*/*R*_0_ is zero when *θ* equals 0° and then reaches the maximum value (0.058 ± 0.0076) when *θ* equals 90°. Notably, the value of Δ*R*/*R*_0_ is not equal to zero when *θ* is 180° due to the stretching force (illustrated by Fig. S7, Supplementary information). Figure 6e shows the NCBEW mechanosensor responding to tilt angles from 0 to 90° with good repeatability. We also found that the NCBEW mechanosensor shows good linearity between tilt angles of 0 and 10° (Fig. 6f). In addition, the inclination angle sensing resolution of the NCBEW mechanosensor is 0.08°, as shown in Fig. 6g, which is higher than some reported flexible tilted sensors^50,51^. The ability of the device to sense tilted angles ranging from −180° to 0° is shown in Fig. S8 (Supplementary information). In summary, we demonstrate that the NCBEW mechanosensor has the function of sensing the inclination angle of the body with respect to the direction of the gravity field, which has not ever been achieved by current biomimetic mechanosensors.

**Fig. 7:**
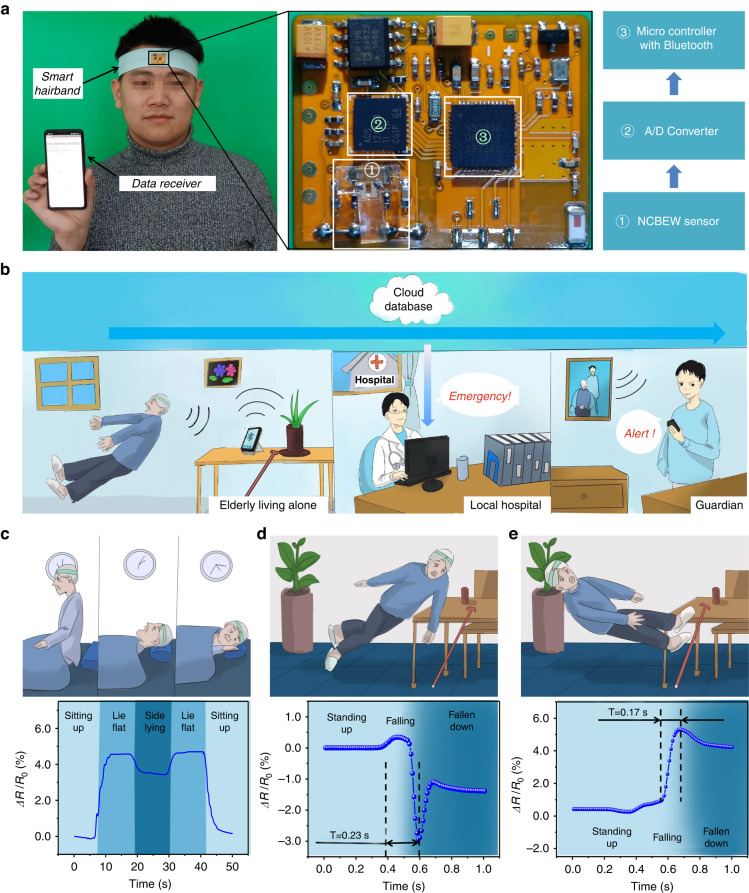
Perception of body orientation with respect to the gravity field and remote monitoring of falls in elderly people. **a** A wearable hairband with a flexible print circuit board consists of an NCBEW mechanosensor, an A/D converter and a microcontroller with Bluetooth. **b** Schematic of the fall alerting system, which monitors elderly individuals via the NCBEW mechanosensor and notifies emergency doctors and guardians when falls occur. **c** Monitoring the daily activities of the elderly via the NCBEW mechanosensor-based smart hairband. **d** The response of the NCBEW mechanosensor-based smart hairband when a person falls forward or (**e**) backward. The event time is used as the criterion to distinguish falls from other daily activities

To achieve a function similar to the human vestibule system, we develop a wearable device that integrates an NCBEW mechanosensor, postprocessing circuits and a Bluetooth unit onto a hairband (Fig. 7a). This system achieves the sensing of the body’s posture and movements and therefore can potentially serve as an artificial vestibule system and has been used for remote monitoring of falls in elderly people.

Falls in elderly people over the age of 65 are prevalent and have become a serious issue for the aging population. Especially for elderly individuals who live alone, if not found in a timely manner, falls can cause injuries, including bone fracture or cerebral hemorrhage, that may lead to paralysis and even death. According to the statistics from the Centers for Disease Control and Prevention in the US, falls among adults 65 and older caused over 32,000 deaths in 2018, making falls the leading cause of injury death for that group^52^. A wearable device with remote monitoring ability is urgently needed to help doctors and guardians take care of the elderly and alert them when emergencies occur. Our device can help to resolve this issue, as shown in Fig. 7b. We developed an elderly fall alert cloud database system. The elderly can wear a smart hairband that contains the NCBEW mechanosensor. A local device such as a smartphone or data router receives the signals from the smart hairband and uploads the data to a cloud database automatically. The data can be shared with doctors in emergency care at local hospitals. At the same time, the guardians of the elderly can also be notified by the cloud database via the smartphone applications we developed.

An elderly person could wear this smart hairband on their head to monitor their sleeping posture (Fig. 7c) and daily activities such as reading, taking medicine, and housework (Fig. S9, Supplementary information). To determine a method that distinguishes falls from other daily activities, we compared the signals when falls occur with those of other activities. Figure 7d, e show the response of the smart hairband when a person falls forward and backward. Spikes are observed when the signal changes rapidly. We define the event time, which equals the duration from the start to the end of the signal change, and use this event time as the criterion to distinguish falls from other activities. The event times for falling forward and backward are 0.23 s and 0.17 s, respectively. The event times for lying down or sitting up are 4.9 s and 4.5 s, respectively (Fig. S10, Supplementary information), which are more than 20 times longer than those of falls. Based on the event time, the system is able to recognize the falls of elderly people and then notify doctors and guardians in a timely manner.

## Conclusion

In this manuscript, a biomimetic mechanosensor is developed by integrating a nanocrack-based strain sensor with a flexible sensing fiber to form a novel electronic whisker. The proposed NCBEW mechanosensor achieves a detection resolution of force as low as 72.2 nN, the perception of tiny objects such as hair airflow, and the recognition of surface morphology down to 30 nm height, which is the finest resolution ever reported regarding biomimetic mechanosensors. In addition to demonstrating the improvements in terms of detection resolution, we utilize the NCBEW device to sense the inclination angle of an object with respect to the Earth’s vertical, which has not been previously achieved by these devices. Therefore, our NCBEW mechanosensor provides a significant advancement in terms of sensitivity and offers great potential to contribute to applications in remote health care.

## Supplementary information

Supplementary information_clean version

supplementary movie
