# Supplementary material for: A nanonewton-scale biomimetic mechanosensor
Source: Microsyst Nanoeng. 2023 Jul 11;9:87. doi: 10.1038/s41378-023-00560-w (PMC10333214; doi:10.1038/s41378-023-00560-w)
Supplement: Supplementary file 1 — Supplementary information_clean version [file 41378_2023_560_MOESM1_ESM.docx]

Supplementary information

A nanonewton scale biomimetic mechanosensor

Chi Zhang‡, Mengxi Wu‡, Ming Li, Lixuan Che, Zhiguang Tan, Di Guo, Zhan Kang, Shuye Cao, Siqi Zhang, Yu Sui, Jining Sun, Liding Wang and Junshan Liu*

‡ Chi Zhang and Mengxi Wu contributed equally to this work.

* Corresponding author.

Email: liujs@dlut.edu.cn (J. Liu)

**This PDF file includes:**

**Note S1** to **S5**

**Figure S1** to **S10**

**Movie S1**

Note S1. Methods

*Fabrication of NCBEW mechanosensor*

The fabrication process of the NCBEW mechanosensor is illustrated as Fig. 1c. First, a smooth 0.5-mm-thick PDMS film (Sylgard 184, Dow Corning Corporation, the weight ratio of the prepolymer and the curing agent was 10:1, and cured at 80 ^o^C for 2 h) is cut into small pieces (30 mm × 20 mm). To facilitate subsequent operation, the PDMS films are put on a polyimide substrate. Then, 5-nm-thick Cr and 50-nm-thick Au layers are sputtered on PDMS successively. Afterward, a layer of positive photoresist (BP212, Beijing Institute of Chemical Reagents, China) is spun onto the Au layer by a spin coater with a speed of 600 rpm for 9 s and 1000 rpm for 30 s. To void the cracking under heating, the soft-baking is conducted at room temperature. The required Au pattern for the electrodes and sensors is defined by a standard photolithography method and chemical etching. In detail, the sample is first immersed into NaOH solution (0.5%), the mixture of I_2_, KI, and H_2_O (1 g: 5 g: 50 mL), and the mixture of H_4_CeN_2_O_3_, HClO_4,_ and H_2_O (10 g: 9 mL: 100 mL) for 30 s, 10 s, and 10 s to remove the exposed photoresist, Au, and Cr. Then the sample is dried by nitrogen and the standard photolithography was used to define the photoresist stripe. After bending the sample with a radius of curvature of 2 mm, nanocracks are obtained. Then the whole pattern is cut as the designed whisker shape with a knife die, in which the length and width of the PDMS cantilever is 8 mm and 2 mm.

The NCBEW mechanosensor with inhomogeneous cross-section is fabricated by shorting the length of the PDMS cantilever to 4 mm, then two pieces of PDMS (3.5 mm × 2 mm) are bonded onto the two sides of the PDMS cantilever after oxygen plasma treatment for 20 s at 20 W.

The encapsulation was conducted by dipping a drop of PDMS on the sensing unit and cured at room temperature for 24h.

*Characterization of the NCBEW mechanosensor*

The morphologies of the samples are characterized by using scanning electron microscopy (SU8220, HITACHI, Japan). The vertical displacement is applied by a universal testing machine (E3000, Instron, USA). The electronic resistance is obtained by using a digital multimeter (PXIe-4081, National Instruments, USA). The noise level is obtained by connecting two electrodes of a NCBEW with two probes of the digital multimeter when no force is loaded on the device. For the study of the effect of the temperature on the response of NCBEW mechanosensors, the temperature change is applied and measured by a temperature controller (2200, Fluke, USA) and a temperature sensor (1502A, Fluke, USA). For the study of the effect of humidity on the response of NCBEW mechanosensors, the humidity change is applied and measured by a humidifier (MJJSQ02LX, Xiaomi, China) and a humidity sensor (DM-1009, DELIXI, China). The heights of photoresist patterns are measured by the surface profilometer (ET4000M, Kosaka Institute, Japan). The speed of the gas flow was measured by a hot-film Anemometer (AR866A, ARCO Science & Technology Ltd, China). The tilt angle was applied by the tunable three-axis stage (DZ-ZDSW-01, Beijing Zhengdong Precision Technology Co., Ltd, China).

Note S2. Electromechanical model of the NCBEW mechanosensor

Taking consideration of the cantilever structure of the NCBEW mechanosensor, the relationship between the strain (*ε*) of the fixed ending and applied force (*P*) at free ending is

| $\varepsilon=\frac{6Pl}{bh^{2}E}=k_{1}P$ | (S1) |
| --- | --- |

Where *l* and E is the length and Young’s modulus of the cantilever, *b* and *h* is the width and thickness of the cross section of the cantilever. The k_1_ is equal to $\frac{6l}{bh^{2}E}$ .

According to previous reports ^S1,S2^, the electromechanical model about nanocrack-based sensors made by Au and PDMS was thought as overlapping effect (*R*_overlap_) and tunneling effect (*R*_tunnel_) as follows:

| $R_{\mathrm{overlap}}=\frac{d\rho_{\mathrm{overlap}}}{l_{\mathrm{overlap}}w_{\mathrm{overlap}}}$ | (S2) |
| --- | --- |

where, *ρ*_overlap_ is the gold interlayer resistivity, *d* is the interlayer distance, *l*_overlap_ is the length of the overlap region, and *w*_overlap_ is the width of the overlap. The width of the overlap region is approximately inversely proportional to the applied strain(*ε*):

| $w_{\mathrm{overlap}}=k_{2}\varepsilon^{-1}$ | (S3) |
| --- | --- |

where, k_2_ is the fitting parameter. Therefore, combined Equation (S1), (S2) and (S3), at the small strain region, the resistance of the device is

| $R_{\mathrm{overlap}}=\frac{d\rho_{\mathrm{overlap}}}{l_{\mathrm{overlap}}k_{2}\varepsilon^{-1}}$ | (S4) |
| --- | --- |

Therefore, the relative resistance change (Δ*R*/*R*_0_, *R*_0_ is the initial resistance) at the stage of overlap effect is

| $\frac{\Delta R}{R_{0}}\approx k_{3}\varepsilon$ | (S5) |
| --- | --- |

And k_3_ is

| $k_{3}=\frac{d\rho_{\mathrm{overlap}}}{R_{0}l_{\mathrm{overlap}}k_{2}}$ | (S6) |
| --- | --- |

According to the previous study ^S1^, at the big strain region, the resistance is

| $\ln\left( \frac{R_{\mathrm{tunnel}}}{R_{0}} \right)=-\ln^{1+\varepsilon}+Xd_{0}\varepsilon$ | (S7) |
| --- | --- |

Therefore, the Δ*R*/*R*_0_ at the stage of tunneling effect is

| $\frac{\Delta R}{R_{0}}=\left( 1+\varepsilon\right)e^{Xd_{0}\varepsilon}-1$ | (S8) |
| --- | --- |

where the *X* is the tunneling barrier height dependent function, *d*_0_ is the average tunneling distance between adjacent crack edges.

As for the strain between the overlap and tunneling, the electrical response was affected by both the overlap and the tunneling effect.

| $\frac{\Delta R}{R_{0}}=k_{4}\varepsilon+k_{5}[\left( 1+\varepsilon\right)e^{Xd_{0}\varepsilon}-1]$ | (S9) |
| --- | --- |

where k_4_ and k_5_ are the fitting parameters.

Note S3. Calculating the maximum strain of the NCBEW mechanosensor

The NCBEW device is regarded as a cantilever. Therefore, the principal strain ($\varepsilon$) is

| $\varepsilon=\frac{M}{WE}$ | (S10) |
| --- | --- |

where *E* is the Young’s modulus, *M* is the bending moment, *W* is the section modulus in bending. While *M* and *W* were

| $M=Pl$ | (S11) |
| --- | --- |
| $W=\frac{bh^{2}}{6}$ | (S12) |

in which, *P* is the concentrated force, *l* is the length of the cantilever, *b* and *h* is the width and the height of the cross-section, respectively.

On the other hand, the deflection ($\omega$) of a cantilever beam under concentrated force (*P*) is

| $\omega=-\frac{Pl^{3}}{3EI}$ | (S13) |
| --- | --- |

And *I* is

| $I=-\frac{bh^{3}}{12}$ | (S14) |
| --- | --- |

Therefore, *F* is

| $P=-\frac{3\omega EI}{l^{3}}$ | (S15) |
| --- | --- |

Combined the these Equations, the principal strain ($\varepsilon$) is

| $\varepsilon=\frac{3\omega h}{2l^{2}}$ | (S16) |
| --- | --- |

Take the height (0.5 mm) and length (8 mm) of the NCBEW device into equation (S16), the strain at the fixed end under the deflection within the range of 0 – 300 μm is 0 – 0.35%.

As for the strain of the nanocrack-based sensing unit of the packaged cantilever under loading of *P* (Fig. S3e), according to equation (S1), the maximum strain is

| $\varepsilon=\frac{6Pl(h_{p}-h_{PDMS})}{b{(h_{p}+h_{PDMS})}^{3}E}$ | (S17) |
| --- | --- |

where, the *h*_p_ and *h*_PDMS_ is the thickness of the packaging materials (PDMS) and PDMS substrate.

The dash line in Fig. S3e indicates the neutral plane of the cantilever, where there is no tensile strain nor compression strain. When *h*_p_ is less than *h*_PDMS_, the nanocrack-based strain sensing unit is stretched, and with the increase of the *h*_p_ the tensile strain decreased, as shown in Fig. S3f. With the further increase of the *h*_p_ (larger than *h*_PDMS_), the nanocrack-based strain sensing unit is compressed and the compressive strain increased. Especially, when the *h*_p_ equal to *h*_PDMS_, the nanocrack-based strain sensing unit is on the neutral plane, and the strain equal to 0.

Note S4. Optimization of the NCBEW mechanosensor with inhomogeneous cross-section sensing fiber

When the tip of the sensing fiber encounters an obstacle such as a bump or a raised stripe, the sensing fiber would bend correspondingly. For a homogeneous sensing fiber, the strain is distributed along the entire fiber. Whereas if the sensing fiber is in-homogeneous, the strain would be concentrated at the points where the thickness is smallest, since the stiffness of the fiber is proportional to reciprocal of thickness. Therefore, we developed a NCBEW device with inhomogeneous cross-section specifically for highly sensitive tactile perception, as shown in Fig. S5a.

Then we compared the strain at the root part of the sensing fiber regarding the homogeneous and inhomogeneous designs. The strain at the root part of the sensing fiber regarding the homogeneous design has been described in Note S3. For the inhomogeneous design, the special sensing fiber is considered as a varying-section cantilever beam. The cantilever beam with the length of *l* and height ranging from $h$ to *βh* is loaded at the free end. As shown in Fig. S5b, the beam can be regarded as two clamped-free sub-beams with different sections. The effective loads at the free end of the thin sub-beam are load *P* and bending moment *M* = *αPl*, in which *α* denotes the length ratio of the thick sub-beam to the whole system.

For a bending-dominated beam, the governing equation is$\frac{d^{2}\omega}{dx^{2}}=M(x)/EI$, in which *ω*, *M*(*x*) and *EI* are deflection, bending moment and bending stiffness, respectively. For the thick sub-beam (grey segment), the boundary condition *ω=0* and *θ=0* at the clamped end gives the deflection${}_{1}$at the free end as ${}_{1}=\alpha^{3}Pl^{3}/3\beta^{3}EI$. For the thin sub-beam (blue segment), the boundary condition *ω=0* and *θ=0* at the clamped end and the load *P* and moment *M* at the free end yields the deflection ${}_{2}$ at the free end is ${}_{2}={(\alpha}^{3}-3\alpha+2)Pl^{3}/6EI$. Moreover, the rotation at the free end of the thin sub-beam yields the following deflection of the thick sub-beam as${}_{3}={(\alpha-\alpha}^{3})Pl^{3}/2EI$. Therefore, the total deflection at the free end of the cantilever system is

| $={}_{1}+{}_{2}+{}_{3}=\frac{Pl^{3}}{3EI}(\frac{\beta^{3}\left( 1-\alpha^{3} \right)+\alpha^{3}}{\beta^{3}})$ | (S18) |
| --- | --- |

Consequently, for a given deflection *ω*_0_ of the cantilever beam, the required load *P*_0_ is estimated as

| $P_{0}=\frac{3{}_{0}EI}{l^{3}(\frac{\beta^{3}\left( 1-\alpha^{3} \right)+\alpha^{3}}{\beta^{3}})}$ | (S19) |
| --- | --- |

For a specified deflection *ω*_0_, the curvature of the thin sub-beam is $\kappa=P_{0}\left(l-x\right)/EI$ and the maximum strain at *x* = 0 can be defined as

| $\varepsilon_{max}=\kappa_{(x=0)}\frac{h}{2}=\frac{3{}_{0}h}{2l^{2}}\frac{\beta^{3}}{\beta^{3}\left( 1-\alpha^{3} \right)+\alpha^{3}}$ | (S20) |
| --- | --- |

Take the height (*h* = 0.5 mm), length (*l* = 4 mm), length radio (*α* = 0.875), thickness radio (*β* = 3) of the NCBEW with inhomogeneous sensing fiber into Equation (S20), the strain at the fixed end under the deflection within the range of 0 – 25 μm was 0 – 0.33%.

Fig. S5c shows the comparison of the strain at the root part of the sensing fiber regarding the homogeneous and the homogeneous designs. The value of strain for inhomogeneous sensing fiber is increased for 1100% than that of the homogeneous design under the same deflection. The response of the NCBEW device with inhomogeneous sensing fiber is also tested when the sensing fiber is loaded with a deflection ranging from 1 μm to 25 μm. As shown in Fig. S5d, the NCBEW device with an inhomogeneous cross-section exhibits a grater response than that of the homogeneous design under the same deflection under the same deflection. Even for the deflection less than 5 μm, the device can distinguish the external load clearly. Therefore, the tactile recognition sensitivity can be improved accordingly.

Note S5. The mechanical analysis of the NCBEW mechanosensor for inclination angle measurement

As shown in Fig. S7, the own weight (*mg*, *m* = *m*_N_ + *m*_H_, *m*_N_ and *m*_H_ is the mass of the neck and the head part of the NCBEW and *g* is the gravitational acceleration) of the NCBEW device would cause the deformation (*ε*) of the fixed ending of the neck. And the deformation changed along with the inclination angle *θ* defined as the angle with the vertical direction. Further, the change of the deformation would produce the change of the resistance of the NCBEW mechanosensor. The *ε* at the fixed ending of the neck was indicated as

| $\varepsilon=\frac{\int_{0}^{(1-\alpha)l} q_{N}tdt+\int_{(1-\alpha)l}^{l} q_{H}tdt}{EI}-\frac{(m_{N}+m_{H})g\cos\theta}{EA}$ | (S21) |
| --- | --- |

in which, *q*_N_=(*m*_N_gsin*θ*)/((1-*α*)*l*), *q*_H_=(*m*_H_gsin*θ*)/*αl*, *E* is the Young’s modulus of the PDMS, *I* and *A* is the moment of inertia and area of the section of the neck, respectively, *α* represents the length ratio as defined in Fig. S5b.


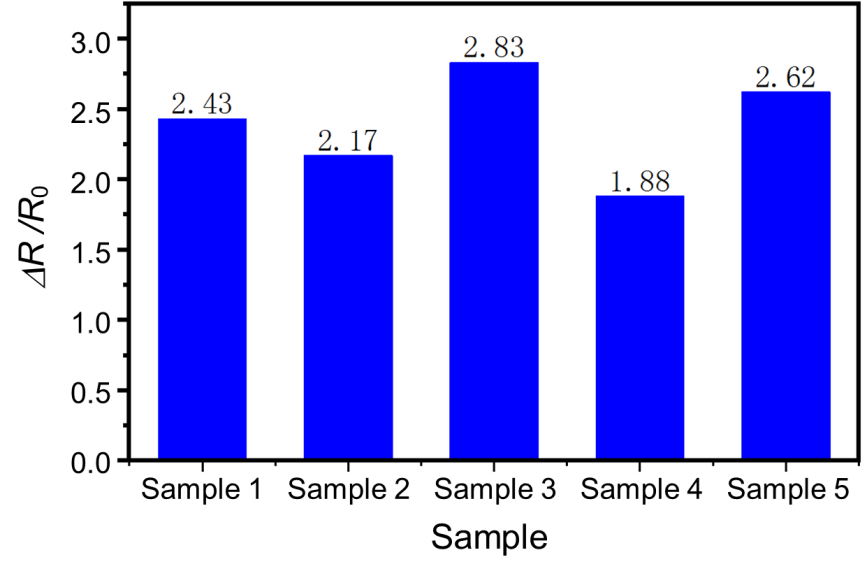


Fig. S1 The responses of five samples under the loading of 67.5 μN.


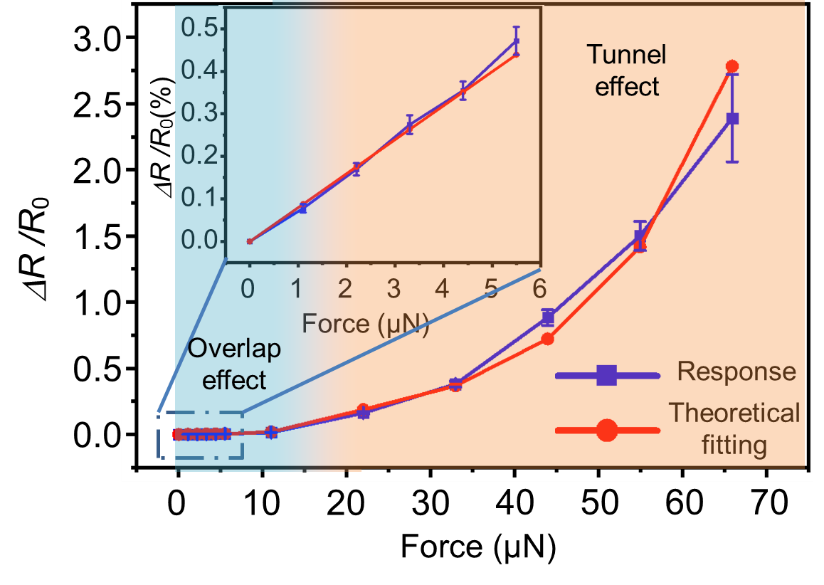


Fig. S2 The theoretical fitting based on the proposed electromechanical model.


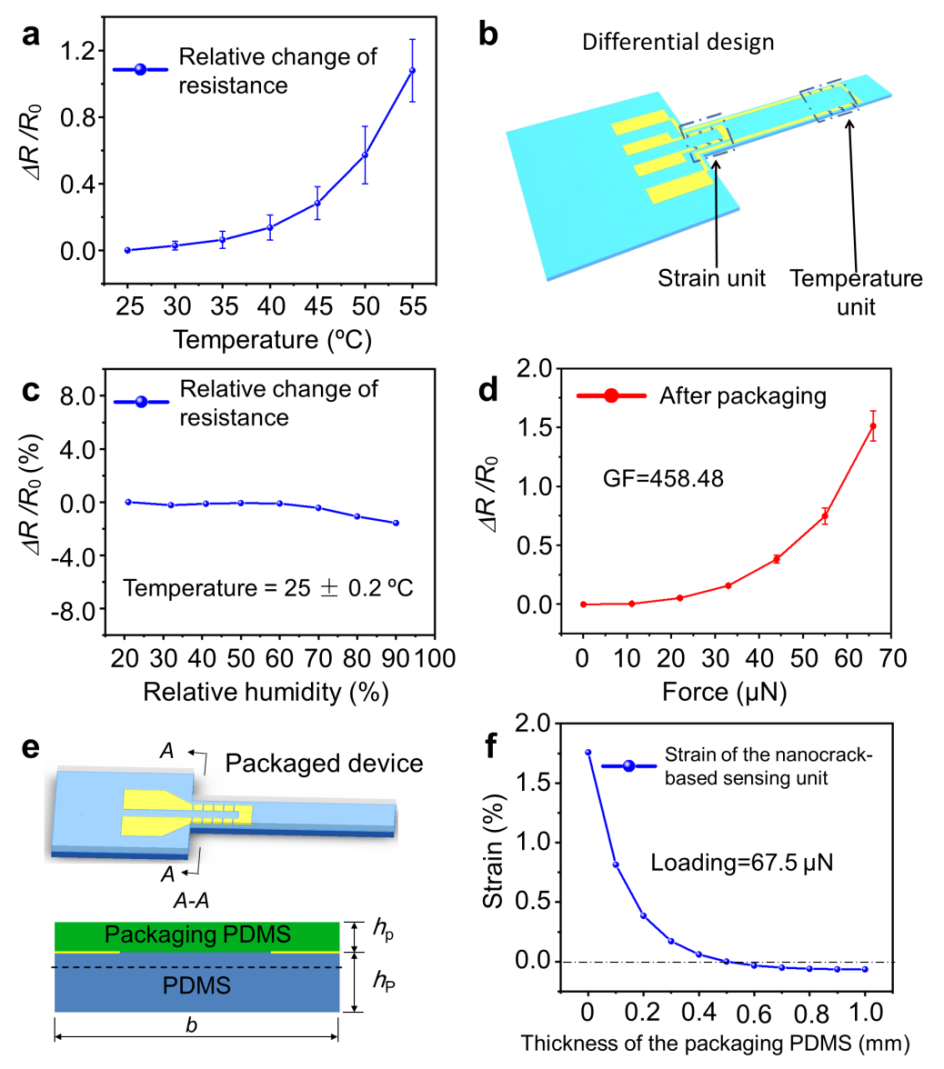


Fig. S3 The effect of temperature, humidity and packaging on the response of the NCBEW mechanosensor. (a) The response of the NCBEW mechanosensor with the temperature changes. (b) A differential design would attribute to decrease the effect of the temperature on the response of the NCBEW mechanosensor. (c) The response of the NCBEW mechanosensor with the humidity changes (the temperature keeps 25 ± 0.2 ^o^C). (d) The response of the NCBEW mechanosensor after packaging. (e) The schematic of the packaged NCBEW mechanosensor, where the dash line indicates the neutral plane of the cantilever. (f) The strain of the nanocrack-based strain sensing unit with the thickness of the packaging PDMS (the loading is 67.5 μN).


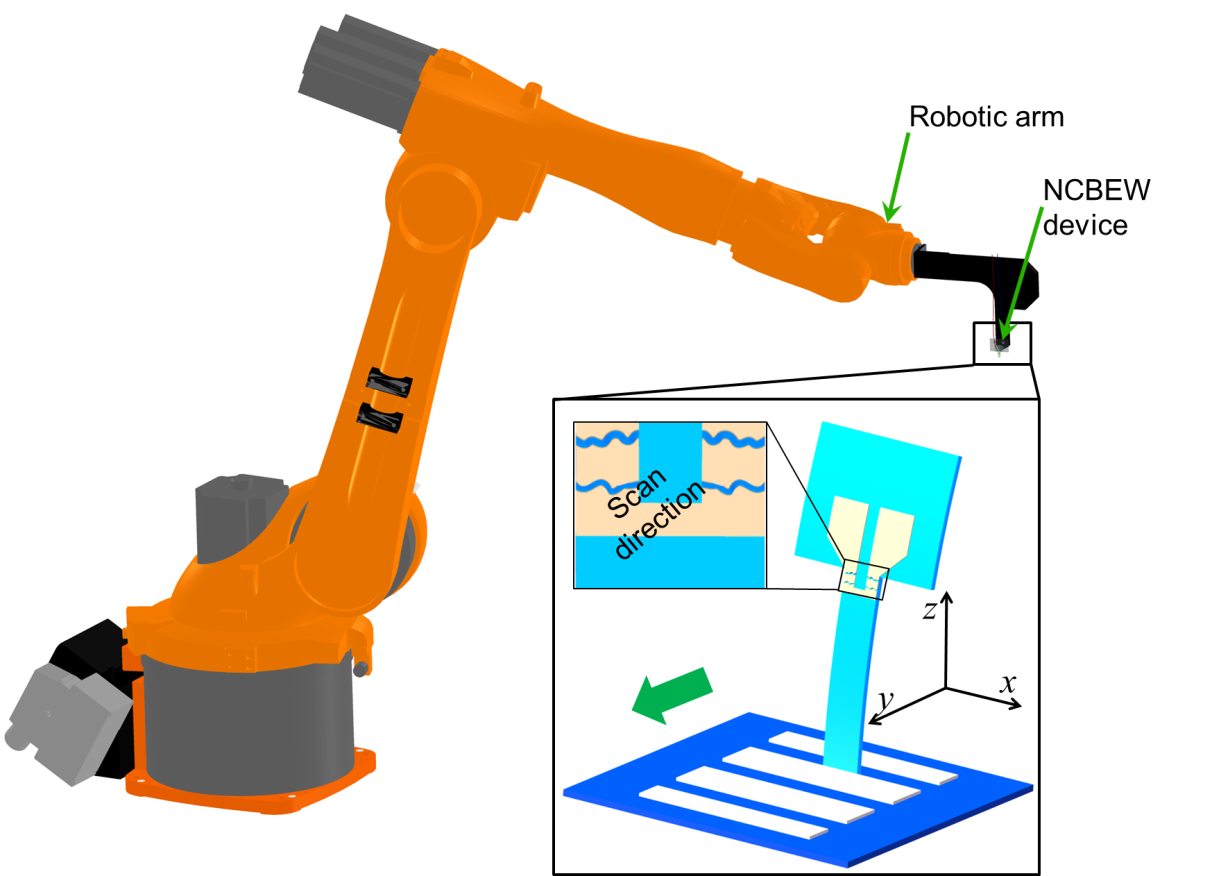


Fig. S4 The NCBEW mechanosensor is attached on the robotic arm for tactile sensing.


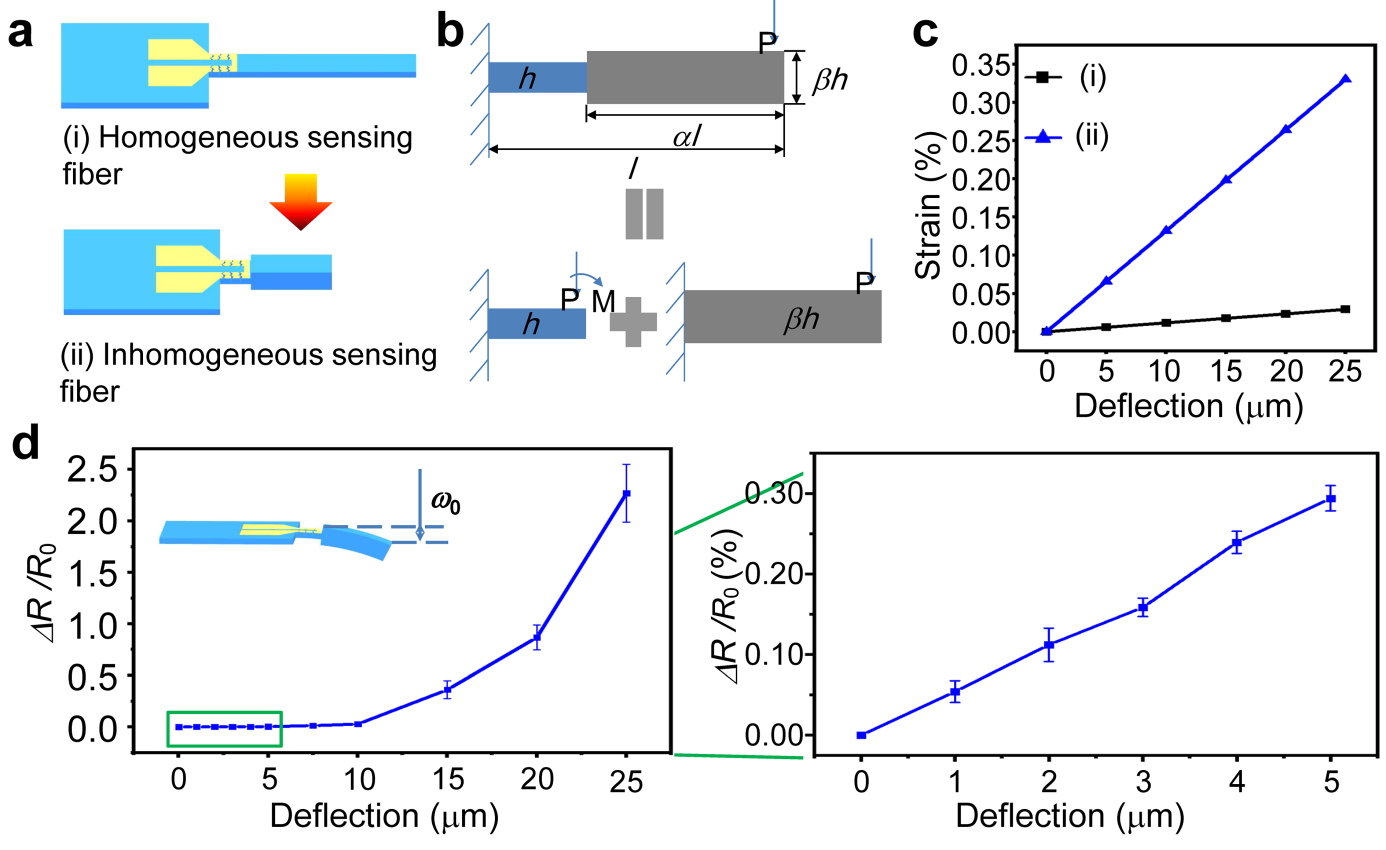


Fig. S5 Optimization of the sensing fiber. (a) Schematic of optimization of the sensing fiber by changing the structure from homogeneous design to a cross-section inhomogeneous design. (b) Mechanical analysis model of the NCBEW device with inhomogeneous design. (c)The comparison between homogeneous sensing fiber and inhomogeneous sensing fiber in terms of the strain at the root part produced by same deflection. (d) The response of the NCBEW mechanosensor with inhomogeneous sensing fiber to deflection ranging of 0 - 25 μm.


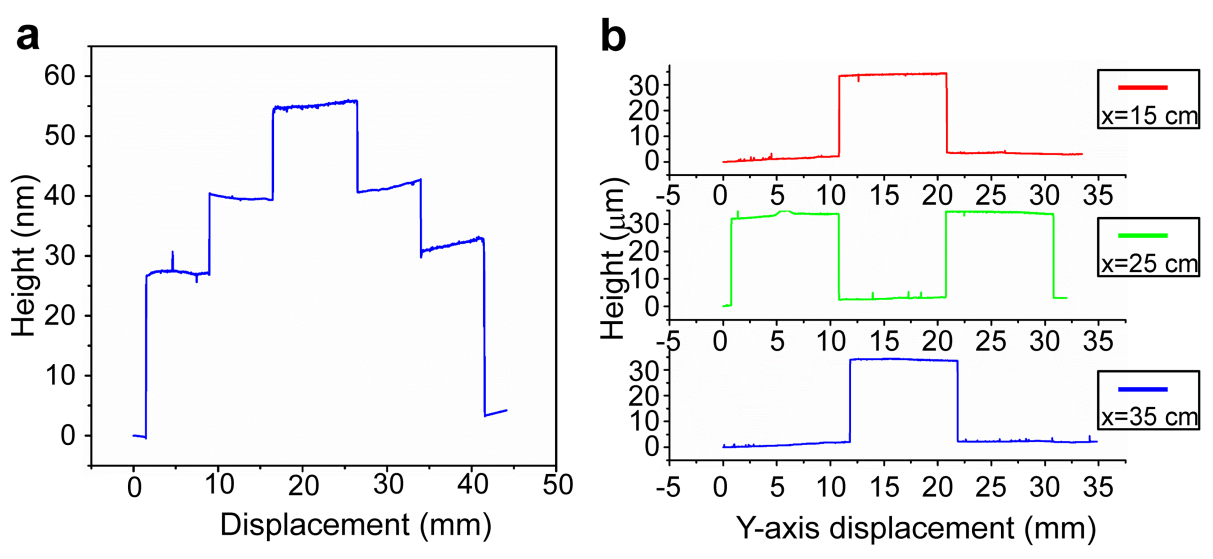


Fig. S6 Surface profilometer measurement results of surfaces. (a) The surface with different-hight stages. (b) The surface with four blocks.


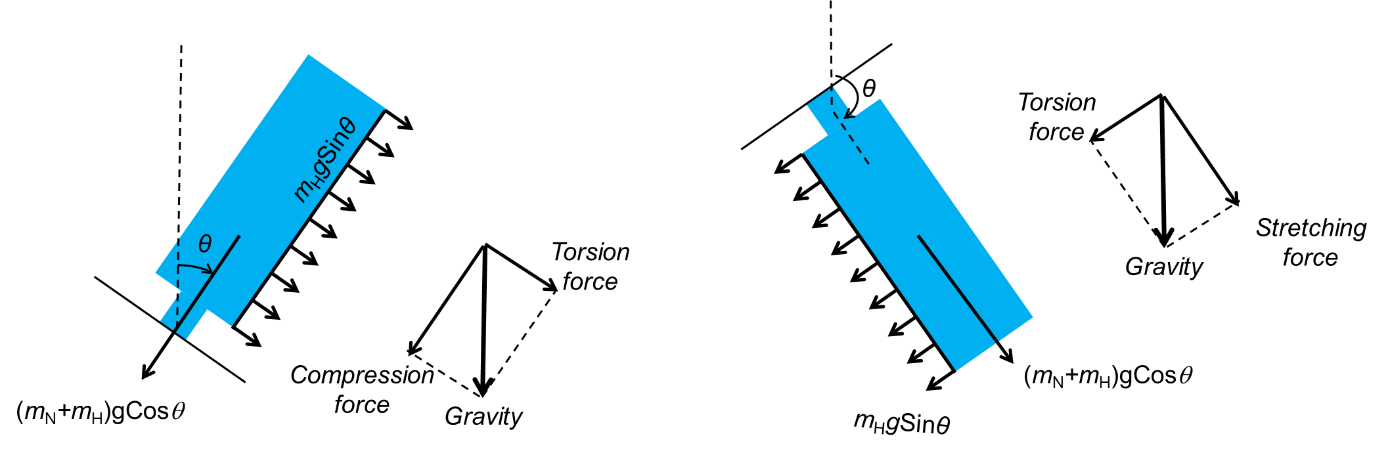


Fig. S7 Mechanical analysis model of the NCBEW mechanosensor for inclination angle sensing.


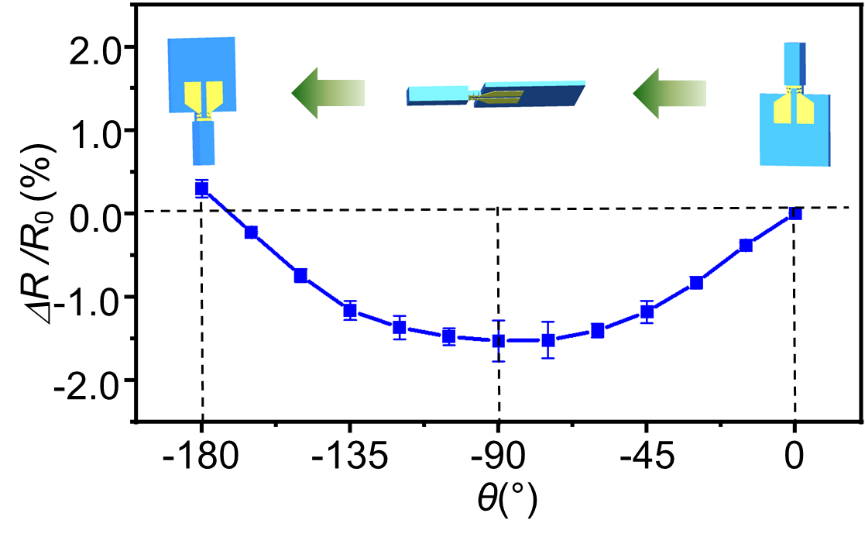


Fig. S8 The performance of the NCBEW mechanosensor in sensing inclination angle ranging from -180 – 0°.


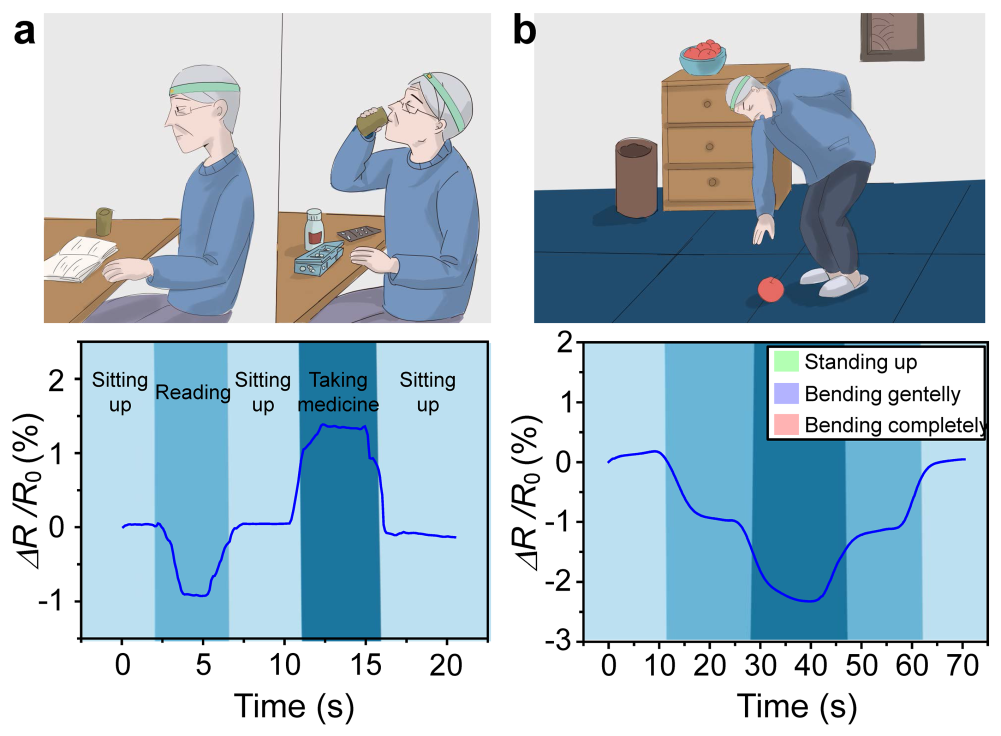


Fig. S9 Monitoring the daily activities via the smart hairband. (a) Reading and taking medicine; (b) picking up.


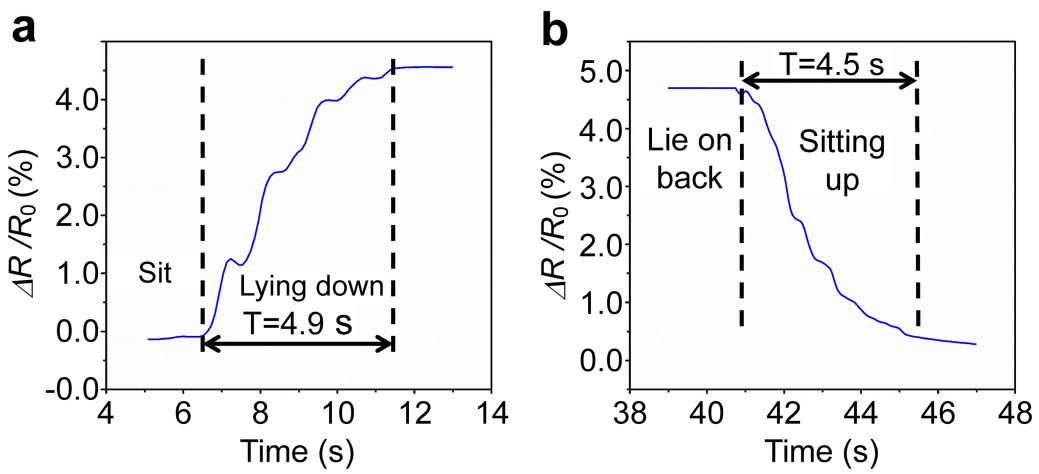


Fig. S10 The event time of for activities including lying down (a) and sitting up (b).


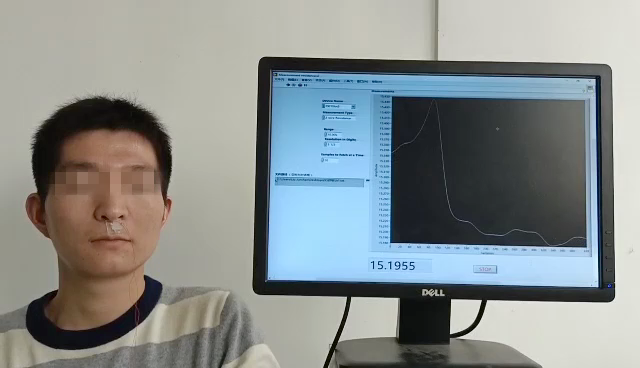


Movie S1. The NCBEW mechanosensor was used to monitor the human breath.

**References:**

S1. Yang, T.T. et al. Structural engineering of gold thin films with channel cracks for ultrasensitive strain sensing. *Mater. Horiz.* **3**, 248-255 (2016).

S2. Wang, C.F. et al. Detection of non-joint areas tiny strain and anti-interference voice recognition by micro-cracked metal thin film. *Nano Energy* **34**, 578-585 (2017).
